# A pilot randomized controlled trial comparing nutritious meal kits and no-prep meals to improve food security and diet quality among food pantry clients

**DOI:** 10.1186/s12889-023-17355-3

**Published:** 2023-12-01

**Authors:** Kelseanna Hollis-Hansen, Carolyn Haskins, Jessica Turcios, Michael E. Bowen, Tammy Leonard, MinJae Lee, Jaclyn Albin, Benaye Wadkins-Chambers, Cynthia Thompson, Taylor Hall, Sandi L. Pruitt

**Affiliations:** 1 Peter O’Donnell Jr. School of Public Health, UT Southwestern Medical Center, 5323 Harry Hines Blvd., Dallas, TX 75390 US; 2 Harold C. Simmons Comprehensive Cancer Center, UT Southwestern Medical Center, Dallas, TX US; 3 Internal Medicine, UT Southwestern Medical Center, Dallas, TX US; 4 Pediatrics and Internal Medicine, UT Southwestern Medical Center, Dallas, TX US; 5 Crossroads Community Services, Dallas, TX US

**Keywords:** Food security, Food assistance, Nutritional sciences, Randomized controlled trial

## Abstract

**Background:**

Food pantry clients have high rates of food insecurity and greater risk for and prevalence of diet-related diseases. Many clients face time, resource, and physical constraints that limit their ability to prepare healthy meals using foods typically provided by pantries. We compared two novel approaches to alleviate those barriers and encourage healthier eating: meal kits, which bundle ingredients with a recipe on how to prepare a healthy meal, and nutritious no-prep meals, which can be eaten after thawing or microwaving.

**Methods:**

Participants were adult pantry clients from a large food pantry in the Southern sector of Dallas, Texas. We conducted a repeated measures between-subjects study with 70 clients randomized to receive 14-days of meal kits (n = 35) or no-prep meals (n = 35). Participants completed questionnaires at baseline and two-week follow-up on demographics, hedonic liking of study meals, perceived dietary quality, and food security. Two-way repeated measures analysis of variance was used to examine group and time effects, and group by time interactions. We also describe feasibility and satisfaction outcomes to inform future implementation.

**Results:**

Sixty-six participants completed the study (94%). Participants were predominantly Hispanic or Latino(a) (63%) and African American or Black (31%) women (90%). There was a significant interaction on hedonic liking of study meals (ηp²=0.16, F(1,64) = 11.78, *p* < .001), such that participants that received meal kits had greater improvements in hedonic liking over time than participants in the no-prep group. We observed significant improvements in perceived dietary quality (ηp²=0.36, F(1,64) = 36.38, *p* < .001) and food security (ηp²=0.36, F(1,64) = 36.38, *p* < .001) across both groups over time, but no between group differences or significant interactions indicating one intervention was more effective than the other. Program satisfaction was high across both groups, but higher among the meal kit group (ηp²=0.09, F(1,64) = 6.28, *p* = .015).

**Conclusions:**

Results suggest nutritious meal kits and no-prep meals may be desirable nutrition intervention strategies for pantry clients and have potential to increase food security and perceived dietary quality in the short-term. Our findings are limited by a small sample and short follow-up. Future studies should continue to test both interventions, and include longer follow-up, objective measures of dietary quality, and relevant clinical outcomes.

**Trial registration:**

This trial was registered on 25/10/2022 on ClinicalTrials.gov, identifier: NCT05593510.

**Supplementary Information:**

The online version contains supplementary material available at 10.1186/s12889-023-17355-3.

## Background

Food pantry clients and members of their households have higher rates of food insecurity and report a greater burden of diet-related conditions and diseases, such as obesity, hypertension, and Type 2 diabetes, than people who do not use food pantries [1–5]. To address these disparities, Feeding America, a United States (US) network of foods banks and pantries, has developed guidelines to improve nutrition in the charitable food system. Guidelines encourage a client choice model that enables clients to select their own food, establishing policies that prioritize fresh produce donations and restrict donations of less nutritious foods, and behavioral economic strategies that promote healthier selections from the pantry (e.g., “nudges”) [6].

For over 10 years our community partner, Crossroads Community Services (hereafter Crossroads), has been at the forefront of implementing Feeding America recommendations [7]. On average, Crossroads serves 20,000 people through their onsite client choice pantry annually. Crossroads refers to the food pantry as a community market and uses the term shoppers instead of clients, to encourage more consistent use of the pantry. The community market offers nutritious inventory, a food selection process created by registered dietitian nutritionists, and supplemental nutrition assistance program (SNAP) coordination onsite. Even with Crossroads implementation of these gold standard procedures and distribution of large quantities of high quality nutritious food, 91% of clients are persistently food insecure (e.g., indicated food insecurity at least 50% of the time within a six-month window) and few clients meet minimum recommended intake for fruits, vegetables, fiber, whole grains, or calcium, while many exceed the maximum intake for added sugar [5]. Previous studies have highlighted a number of barriers to healthier food preparation and consumption among people that use food pantries: high costs associated with healthier foods, lack of kitchen space and tools for food preparation, chronic health conditions or disabilities that make food preparation physically challenging, and lack of time [8–10]. Taken together, these findings suggest additional nutrition intervention strategies are needed to effectively increase food security, improve dietary quality, and ultimately reduce diet-related disease among pantry clients.

Meal kits and no-prep meals are two burgeoning food retail strategies [11, 12] that could be developed into nutrition interventions that facilitate healthier eating among people that use food pantries, as these strategies may help clients’ overcome the aforementioned barriers to healthier eating. Meal kits pair fruits, vegetables, whole grains, and lean proteins with a recipe that instructs participants on how to cook the meal [13]. Most often, meal kits provide unprepared food ingredients that require some preparation before cooking, however, there is currently no strict definition of a meal kit, and the amount of preparation needed to prepare a meal kit may vary. For example, some commercially available meal kits require cleaning and cutting of produce prior to cooking, while others may provide precut or frozen produce. In studies of grocery shoppers, meal kits that provide unprepared, bundled ingredients increase perceived value of goods [14] and compared to selecting individual ingredients, are hypothesized to reduce search and planning time associated with making healthier food choices [15]. Meal kits were reported as a preferred nutrition strategy by pantry clients in a previous research study [16] and were found to increase selection of kale and whole grains when compared to recipe tasting and “treatment as usual” control groups in a between-subjects experiment in Connecticut [17].

Nutritious no-prep meals are prepared meals that are lower-calorie, lower-sodium, and lower-sugar than most commercially available no-prep meals and often contain a higher volume of nutritious foods (e.g., produce, whole grains, lean proteins). These meals can safely be eaten after thawing, however, reheating in a microwave or oven is recommended for optimal taste and consistency. No-prep meals alleviate time and food preparation constraints faced by people experiencing food insecurity and provide the opportunity to encourage more nutritious food consumption in a potentially preferable package. No-prep meals also provide a use for food products that are near the end of their shelf-life whereas meal kits must include items that can last until the recipient is ready to use them. No-prep meal production, at scale, can provide inventory management advantages. Some food pantries and food banks already provide similar meal programs. For example, the Central Texas Food Bank is one of many that use their commercial kitchen to repurpose fresh produce and dry good donations into frozen meals for seniors [18]. Empirical research is needed to understand client preferences for meal kits and no-prep meals and to determine whether these programs improve food security and diet quality.

Client choice, taste preference, participant satisfaction, and program feasibility are imperative to the success of nutrition interventions [19, 20]. In this pilot randomized controlled trial, we measure hedonic liking of study meals, participant satisfaction, and feasibility of these cutting-edge nutrition interventions to inform future implementation. We also measure food security and perceived dietary quality to test if providing meal kits and no-prep meals in addition to typical pantry provisions have potential to increase food security and dietary quality. This study is the first of its kind and will provide critical information on how to carry out these food distribution programs for food pantry clients. Our preliminary hypotheses were that both groups would have improvements in hedonic liking of study meals, perceived dietary quality, and food security over the study period, and that participants in the group receiving no-prep meals would have greater improvements than those receiving meal kits, given that no-prep meals reduce burdens associated with food preparation.

## Methods and analysis

This study was approved by the UT Southwestern Medical Center Institutional Review Board (Identifier: **STU-2022-0809**). A step-by-step description of the study protocol is available in Supplementary Material 1.

### Recruitment

Clients were recruited from February 12th -April 4th, 2023, by study staff who sat at a table with study signage in the middle of Crossroads waiting room during open food distribution hours (Monday-Thursday 8:30 AM–1:30 PM, first Saturday 8:30–11:30 AM). If clients were not approaching the table, staff would make a brief announcement to let clients know about the study. Fliers were also present throughout the pantry and clients could scan a QR code on the flier to complete the eligibility screener if study staff were with other participants.

### Eligibility

Study inclusion criteria included adults 18 or older; able to read, write, and/or speak English or Spanish fluently; able to provide informed consent; clients who had used Crossroads pantry at least once; and able and willing to participate in the study, which included willingness to return in two-weeks to complete the follow-up appointment. Study exclusion criteria were dietary restrictions, allergies, or sensitivities that could put the participant at-risk of harm from consuming study foods. Some restrictions could be accommodated (e.g., vegetarian), while strict dietary needs (e.g., ketogenic diet, vegan diet), dietary disorders (e.g., celiac disease), and food allergies (e.g., dairy) were excluded as we could not guarantee no cross-contamination between study foods and products that could trigger an allergy or illness. Five people out of the 102 screened reported one of these dietary restrictions (5%). Clients were screened for eligibility by completing a 10-item survey on a study tablet or from their electronic device. Staff assisted clients uncomfortable with the tablet by reading questions and entering responses. The eligibility survey took an average of five minutes.

### Power

This was the first of its kind pilot study, therefore there were no a priori effect sizes to calculate a power analysis. Using G*Power 3.1, we determined a small to medium size between-within interaction effect (f = 0.17) can be detected with a sample of 70 participants, thus we planned to stop enrollment at 70 clients.

### Study procedures

#### Consent process

After eligibility was confirmed, clients could choose to participate in the first appointment immediately or to schedule the appointment for a future date. At the first appointment clients were read the consent form in English or Spanish and were asked if they had questions about the study. Once questions were answered or if there were no questions, clients were consented to participate. The time and date of verbal consent were recorded by study staff. Clients were consented and enrolled in the study by authors CH and JT.

#### Baseline measures

Participants completed a baseline questionnaire which included questions on demographics (age, race/ethnicity, adults and children in the home, annual income, and years of education). Two-items on medical insurance coverage were asked “*Was there any time during the past two years when you did not seek medical care because it was too expensive, or health insurance did not cover it*?” and “*In the past two years have you always had health insurance or other medical coverage for health care?*” which had Yes/No responses [21]. To measure food security we used the United States Department of Agriculture (USDA) six-item household food security scale which was coded as a continuous variable (0–6, high to very low food security) for analysis of variance and a categorical variable for participant characteristics (0–1 = High or marginal food security, 2–4 = Low food security, 5–6 = Very low food security) [22]. Perceived diet quality was measured using a single validated item that asked participants to rate their diet as “excellent” “very good” “good” “fair” or “poor” (5 = Excellent, 1 = Poor) [23].

#### Randomization

Participants were randomized by first author (KH) using simple randomization by generating random study identification numbers and randomly assigning those identification numbers to Group 1 (Meal kits) or Group 2 (No-prep meals) using a 1:1 allocation ratio. Assignments were concealed until the participant had to select study foods, at which point there was no way to conceal assignment.

#### Hedonic liking measurement

The questionnaire was programmed to bring up questions on hedonic liking that aligned with the participants group assignment; participants in Group 1 saw pictures of meal kits, while participants in Group 2 saw pictures of no-prep meals. However, participants were not told they were answering questions pertaining to their group assignment and thus were unlikely to be aware of their assignment at this point. This section of the questionnaire was also programmed to randomize question order (e.g., the order in which meals were shown) to reduce the likelihood of an order effect. Hedonic liking of study meals was measured by showing the name and picture of the meal and asking participants to respond to a 9-point bipolar scale with four measures of liking, four measures of dislike, and a neutral item, higher scores indicated higher hedonic liking [24].

#### Selection of study meals and nutritional content of meals

After participants finished the hedonic liking questionnaire, they were presented with laminated cards that displayed pictures of meals available depending on the participants group assignment. In both groups clients were able to select up to 84 servings of study meals as it was enough for a household of three (the average household size in Dallas County) [25] to have two meals per day each day of the two week study period. Meal kits were matched in quantity and content as closely as possible to the no-prep meals, such that a meal kit would include recipes and ingredients equivalent to three servings of a no-prep meal. We also aimed to replicate the no-prep meals standard weight of meat and grains as closely as possible when creating recipes and selecting ingredients for the meal kits. Participants were able to select from a menu of 14 breakfasts and 14 dinners and could select up to six servings or two meal kits of each available meal as an inventory control measure. Table 1 provides a brief description of each meal and the average nutritional content of each meal across groups. Nutritional content for the no-prep meals came directly from the no-prep meal distributor. Axxya Nutritionist Pro™ v7.9 software was used to conduct nutritional analysis of each meal kit. Ingredients in the meal kits had a mix of perishable (e.g., carrots, cheese, whole wheat bread), semi-perishable (e.g., potatoes, precooked chicken), and non-perishable (e.g., brown rice, low-sodium black beans, frozen produce) items. A full description of the ingredients in each meal kit is in Supplementary Material 2. Clients indicated food selections to a study staff member who entered the selections into an excel spreadsheet that calculated the total number of items and servings selected.

**Table 1:** Nutrient composition of meal kits and no-prep meals

		Meal kits		No-prep meals		
	Nutrients	Mean ± SD	Min-Max	Mean ± SD	Min-Max	*p*-value
Breakfast Entrees	Calories	263 kcal ± 74.9	138–349	229 kcal ± 53.5	130–310	0.170
	Saturated fat	1.9 g ± 1.6	0.1–4.4	2.4 g ± 2.2	0–5	0.511
	Sodium	433 mg ± 187	276–830	449 mg ± 85.5	330–620	0.766
	Carbohydrates	30.4 g ± 15.5	13–59	29.1 g ± 9.2	15–39	0.803
	Fiber	**9 g** ± **5**	**1–20**	**2.7 g** ± **1.3**	**1–6**	< 0.001***
	Sugar	4.5 g ± 2.7	2–12	3.1 g ± 2.1	2–10	0.134
	Protein	**17.6 g** ± **6.9**	**9–33**	**13 g** ± **1.8**	**11–18**	0.024*
Lunch/ dinner Entrees	Calories	409 kcal ± 73.1	262–513	338 kcal ± 102	160–460	0.054
	Saturated fat	**1.1 g** ± **0.5**	**0.4–1.8**	**3.6 g** ± **2.2**	**1–7**	< 0.001***
	Sodium	542 mg ± 241	157–908	371 mg ± 147	80–600	0.041
	Carbohydrates	**46.9 g** ± **6.4**	**39–74**	**33.6 g** ± **5.4**	**21–45**	< 0.001***
	Fiber	**7.1 g** ± **1.6**	**5–10**	**4.2 g** ± **1.5**	**1–8**	< 0.001***
	Sugar	**8.9 g** ± **2.8**	**4–13**	**6.8 g** ± **2.3**	**4–11**	0.040*
	Protein	21.4 g ± 7.7	7–34	16.9 g ± 9.4	5–31	0.191

*p* < .05, **=*p* < .01, ***=*p* < .001; These results are for the nutrient composition of the meals as provided, they do not include any changes made by participants (e.g., seasonings)

While clients went through Crossroads typical ordering procedures with pantry staff, study staff collated the participants study selections. Study staff were trained on safe food storage and handling practices by Crossroads staff prior to the onset of the study and followed all food safety guidelines set forth by Feeding America, which are inclusive of and more stringent than rules governing grocery retailers, food manufacturers and restaurants in the US [26]. No-prep meals were retrieved from a walk-in freezer and brought to the participant. Ingredients for meal kits were bundled in a recyclable paper bag and a recipe was stapled or taped onto the outside of the bag to indicate how to prepare the meal. An electronic copy of the recipes was provided by email to participants in the meal kit group upon request. Clients were instructed that no-prep meals could be stored in a refrigerator or freezer and eaten after thawing or reheating depending on consumer preference.

#### Crossroads typical pantry procedures

Clients can visit Crossroads pantry once each month and select food for up to 21 meals for each person in their household. Clients select food with a pantry staff member on a computer with a live Salesforce inventory system (Salesforce Inc., San Francisco, CA). The Salesforce interface uses a point system created by registered dietician nutritionists, that considers the age, gender, and activity level of each member of the household and indicates how many of each type of food group clients can select based on their nutritional needs. Clients are told how many points they have, and each available food item has a point value. After clients make food selections, their list is printed, and they take a grocery cart through the physical pantry aisles, like a grocery store. At checkout, pantry provisions are bagged, and a volunteer assists the client to their vehicle. Participants followed these same procedures during the study, except that study staff and/or student research assistants met the clients at pantry checkout and helped the participant load their vehicle, at which point clients were able to ask any questions and were reminded of the date and time of their follow-up appointment.

#### Follow-up questionnaire

Participants completed the same hedonic liking, perceived diet quality, and food security measures at follow-up in-person, except the wording was slightly modified to the temporal window (e.g., “*How would you rate your dietary quality ****over the past 2-weeks****?*”). Participants were also asked questions on intervention satisfaction adapted from prior studies with pantry clients: “*The food has been helpful*” “*The food provided was food my household likes to eat*” “*The food provided was good quality*” “*Enough food was provided*” “*I know how to prepare the foods*” with “Strongly agree” “Agree” “Disagree” and “Strongly disagree” response options [27]. The items were summed to create an Intervention Satisfaction variable with the lowest possible score of 4 if all responses were “Strongly disagree” and the highest possible score of 20 if all responses were “Strongly agree.”

To measure intervention fidelity, we asked clients in both groups “*Thinking about the times when you ate the study meals. Did you add additional ingredients or eat additional food when eating the study meals?*” and clients were instructed to select all of the following options that applied: “Yes, I added extra seasoning or condiments (e.g., hot sauce, ketchup, mustard, salt, pepper, etc.)” “Yes, I added extra food items *into* the meal (e.g., I mixed extra meat, egg, protein, vegetables, fruit, etc. into the meal)” “Yes, I ate separate food or snacks in addition to the meal” *or* they could indicate “No, I ate the meal as is” as a single response. We asked, “When thinking about the size or quantity of the food in each study meal, was the size…” with response options “Too little (not enough food)” “Just right (the right amount of food)” and “Too big (too much food),” we asked for breakfast and lunch/dinner meals separately. Participants that received meal kits were asked “Did you use the recipe provided on the recipe card attached to each meal kit?” with Yes/No options. Participants were also asked “*Over the past year, how many months have you gotten food from this food pantry*” and could select up to 12 from a dropdown menu, indicating they had come every month within the past year.

#### Missed appointment protocol

If a participant missed the follow-up appointment, we had a protocol to contact the participant using their preferred method of contact twice on the day of the missed appointment and once per day for the two days following the appointment. Thereafter, we would contact once per week for the next four weeks.

#### Intervention costs

We added the total spent to purchase all the ingredients and supplies needed to create the meal kits and the total amount spent to purchase no-prep meals and divided those numbers by the number of meals provided in each group. Half of the no-prep meals were generously donated by the distributor; therefore, we calculated costs with and without the donation.

### Analytic plan

IBM SPSS Statistics for Windows, Version 28.0. Armonk, NY: IBM Corp was used to conduct data analyses. Unpaired t-tests were used to determine differences between nutrient composition by group. Descriptive statistics (e.g., means, percentages, chi-squared tests of independence) were used to describe participant characteristics, categorical baseline differences, and costs associated with purchasing each study meal. Fisher’s exact tests were used if categorical items had small cell counts (e.g., gender, items on size/quantity of meals). One way analysis of variance (ANOVA) was used to test baseline differences in continuous variables (e.g., age, education) and number of servings selected by group. Distributions and QQ plots were checked. Two-way repeated-measures ANOVA was used to test for group and time effects on study outcomes of interest (hedonic liking of study meals, perceived diet quality, food security) and group-by-time interactions. Bonferroni correction was used for analysis on the hedonic liking of study meals as assessing differences by group over time for each meal leads to 28 comparison tests and high risk for Type 1 error. Therefore, Bonferroni adjusted α is set at 0.00179 for each individual meal, meaning the null hypothesis should only be rejected if the *p*-value is < 0.00179. One way ANOVA was used to test for follow-up differences in continuous variables (e.g., Intervention Satisfaction).

## Results

### Enrollment

Of clients that completed the eligibility screener (n = 102), 70 enrolled in the study (69%), and 32 were excluded. Six clients were ineligible, one reported celiac disease and five were not willing or able to return to the pantry for follow-up. Twenty-two clients declined to participate but did not provide a reason. Four clients decided to enroll in a separate cross-sectional survey study we were conducting concurrently instead, which did not include randomization or receipt of study meals (participants were not able to participate in both studies). Forty-nine clients participated the same day they completed the eligibility screener (70%), and 21 clients scheduled the baseline appointment for a future date (30%).

### Retention

**Fig. 1:**
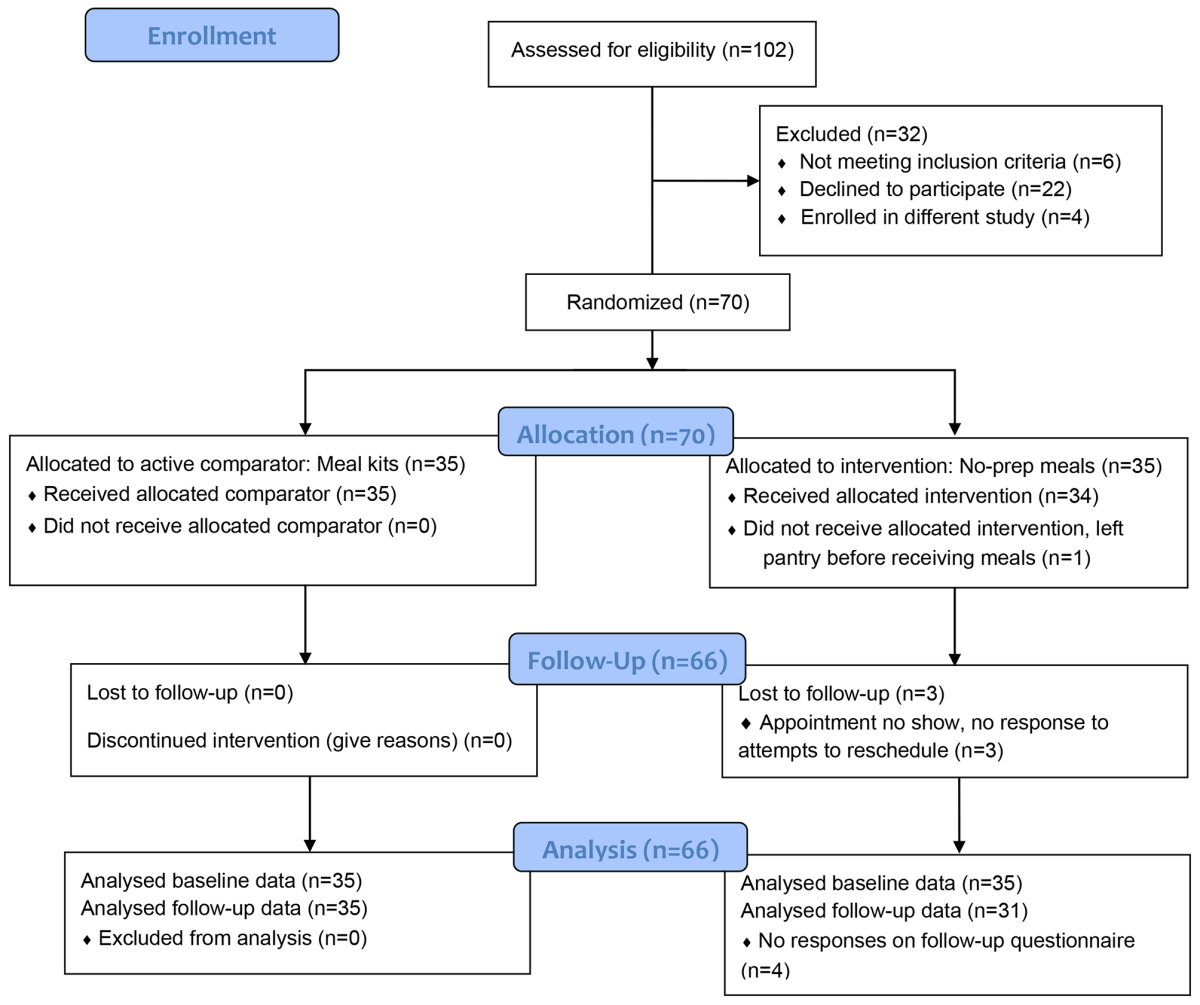
CONSORT 2010 Flow Diagram Provides a CONSORT 2010 flow diagram detailing study participation from eligibility screening to follow-up

Of the 70 people who enrolled, consented, completed baseline questionnaires, and were randomized, 69 received their allocated intervention. One participant in the no-prep group left the pantry before receiving their meals and did not respond to attempts to contact. While we had to reschedule appointments to accommodate changing schedules, only three participants were lost to follow-up and there was an average of 15.6 days between baseline and follow-up across participants (Min = 14, Max = 34). Sixty-six participants completed all study procedures (94%), which included all 35 participants randomized to the meal kit group (100%) and 31 participants randomized to the no-prep group (89%). Figure 1 is a CONSORT Flow Diagram.

### Participant characteristics

Most participants were female (90%), Hispanic or Latino (63%) and African American or Black (31%). On average, participants were 58.6 years old ± 13.6 years, had an annual household income of $19,058 ± $9,277 USD, and a household size of 3.7 people ± 2.2 people. 47% of participants did not seek medical care at some point within the past two years due to cost and 47% did not have medical insurance at some point within the past two years. On average, participants had visited Crossroads food pantry 6.61 months ± 3.95 months out of the past 12 months. There were no group differences for any participant characteristics across study arms, except years of education. Mean years of education of participants that received no-prep meals was 2.3 years higher than participants that received meal kits (F(1,66) = 7.26, *p* = .009). Participant characteristics are provided in Table 2.

**Table 2:** Participant Characteristics

	Across participants (N = 70)	Group 1 Meal Kits (N = 35)	Group 2 No-prep (N = 35)	Difference between groups	
**Race**				0.337	
African American/Black	22(31%)	10(29%)	12(34%)		
American Indian/Alaska Native	1(1%)	1(3%)	0(0%)		
Hispanic/Latino	44(63%)	24(69%)	20(57%)		
Multiracial	2(3%)	0(0%)	2(6%)		
White	1(1%)	0(0%)	1(3%)		
**Preferred language**				0.053	
English	40(57%)	16(46%)	24(69%)		
Spanish	30(43%)	19(54%)	11(31%)		
**Gender**				0.500	
Female	63(90%)	32(91%)	31(89%)		
Male	7(10%)	3(9%)	4(11%)		
**Have Medical Insurance**	37(53%)	19(54%)	18(51%)	0.811	
**Did Not Seek Medical Care When Needed**	33(47%)	14(40%)	19(54%)	0.231	
**Food Security**				0.221	
High/marginal food security	18(26%)	8(23%)	10(29%)		
Low food security	33(47%)	20(57%)	13(37%)		
Very low food security	19(27%)	7(20%)	12(34%)		
**Income (US Dollars)**	19,058 ± 9277	20,220 ± 9988	17,928 ± 8521	0.308	
**Education (Years)**	**11.51 ± 3.67**	**10.33 ± 3.77**	**12.63 ± 3.25**	0.009**	
**Age (Years)**	58.57 ± 13.62	60.43 ± 13.71	56.71 ± 13.48	0.257	
**Household Size (People)**	3.70 ± 2.19	3.71 ± 2.28	3.69 ± 2.13	0.957	
**Pantry Utilization (Months)**	6.61 ± 3.95	6.43 ± 4.38	6.81 ± 3.46	0.701	

Values are n(%) for categorical variables and mean ± standard deviation for continuous variables; *=*p* < .05, **=*p* < .01, ***=*p* < .001

### Intervention outcomes

Participants in the no-prep group selected an average of 66 servings and participants in the meal kits group selected an average of 20 kits or 60 servings; there were no significant differences in servings selected by group (F(1,69) = 1.38, *p* = .24). Similarly, there were no significant differences in servings selected per person in the household by group (F(1,69) = 0.05, *p* = .83). Participants in the meal kit group selected an average of 25 servings per member of the household and participants in the no-prep group selected an average of 26 servings per member of the household. Supplementary Material 3 includes a table with servings selected by household size and servings selected by number of people in the household.

**Fig. 2:**
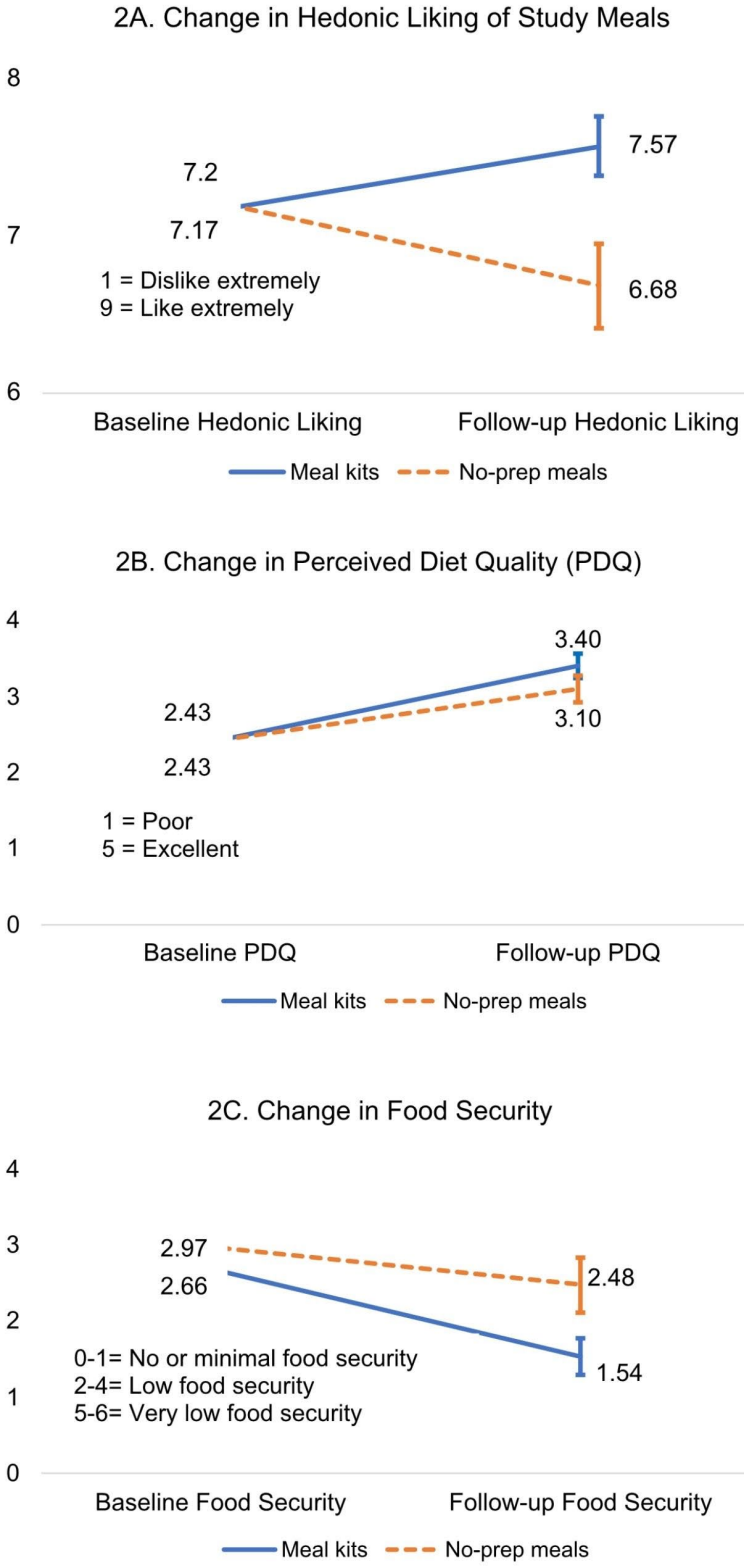
For all figures the solid blue line represents the group that received meal kits and the dotted orange line represents the group that received no-prep meals. Error bars indicate the standard error of the group mean. (**A**) displays the change in hedonic liking of study meals from baseline to follow-up by group. There was a significant interaction on hedonic liking of study foods. Participants that received meal kits had greater improvements in hedonic liking of study meals over time (M = 7.57, SE = 0.19, Min = 4, Max = 9) than participants in the no-prep group (M = 6.68, SE = 0.27, Min = 2.32, Max = 9). (**B**) illustrates participants change in perceived diet quality from baseline to follow-up by group. Both groups reported their diet was higher quality at follow-up than at baseline. Participants that received meal kits had slightly higher perceived diet quality at follow-up (M = 3.40, SE = 0.16, Min = 2, Max = 5), than participants that received no-prep meals (M = 3.10, SE = 0.18, Min = 1, Max = 5), however, this was not a statistically significant difference between groups and no interaction was observed. (**C**) displays participants change in food security from baseline to follow-up by group. Both groups reported higher food security (lower food insecurity) at follow-up than at baseline. Participants that received meal kits had slightly higher food security (lower food insecurity) at follow-up (M = 1.54, SE = 0.24, Min = 0, Max = 6), than participants that received no-prep meals (M = 2.48, SE = 0.36, Min = 1, Max = 5), however, this was not a statistically significant difference between groups and no interaction was observed

There was a significant interaction on hedonic liking of study foods (ηp²=0.16, F(1,64) = 11.78, *p* < .001), such that participants that received meal kits had greater improvements in hedonic liking of study meals over time (7.6 ± 1.1, Min = 4, Max = 9) than participants in the no-prep group (6.7 ± 1.5, Min = 2.32, Max = 9). A description of study meals, and the average hedonic liking of each study entrée from baseline to follow-up is presented in Table 3. Fig. 2A displays the change in hedonic liking scores from baseline to follow-up by group and the interaction.

**Table 3:** Hedonic Liking of Study Meals

		Across Participants		Group 1 Meal Kits		Group 2 No-prep		*p*-value
	Entrée	Baseline N = 70	Follow-up N = 66	Baseline N = 35	Follow-up N = 35	Baseline N = 35	Follow-up N = 31	
All Entrees	Average rating across all entrees	7.2 ± 1	7.2 ± 1	7.2 ± 1.1	7.6 ± 1.1	7.2 ± 0.9	6.7 ± 1.5	0.001***
Breakfast Entrees	Average rating across breakfast entrees	7.2 ± 1	7.3 ± 1.3	7.2 ± 1	7.5 ± 1.2	7.3 ± 1	7.1 ± 1.4	0.113
	Turkey sausage bowl^$^	7.5 ± 1.6	7.6 ± 1.5	7.8 ± 0.8	7.7 ± 1.4	7.3 ± 2	7.4 ± 1.5	0.905
	Buttermilk waffles	7.4 ± 1.4	7.4 ± 1.7	7.3 ± 1.3	7.3 ± 1.7	7.6 ± 1.5	7.5 ± 1.8	0.725
	Turkey sausage burrito^$^	7.4 ± 1.6	7.6 ± 1.5	7.5 ± 1.4	7.7 ± 1.4	7.3 ± 1.7	7.6 ± 1.6	0.899
	Blueberry waffles	7.4 ± 1.7	7.5 ± 1.6	7.4 ± 1.2	7.5 ± 1.6	7.5 ± 2.1	7.4 ± 1.7	0.824
	Cinnamon french toast	7.3 ± 1.3	7.6 ± 1.5	7 ± 1.4	7.7 ± 1.3	7.7 ± 1.2	7.5 ± 1.8	0.054
	Southwestern veggie burrito	7.3 ± 1.5	7.1 ± 1.8	7.1 ± 1.3	7.3 ± 1.5	7.4 ± 1.7	6.8 ± 2.1	0.033
	Spinach and mozzarella flatbread^$^	7.3 ± 1.7	7.2 ± 1.7	7 ± 1.7	7.3 ± 1.6	7.5 ± 1.7	7.1 ± 1.8	0.059
	Fire roasted salsa burrito^$^	7.2 ± 1.4	7.1 ± 1.9	7.1 ± 1.5	7.4 ± 1.7	7.3 ± 1.4	6.8 ± 2.2	0.251
	Three cheese flatbread^$^	7.2 ± 1.4	7.3 ± 1.6	7.1 ± 1.4	7.3 ± 1.7	7.3 ± 1.3	7.3 ± 1.5	0.613
	Spinach scramble burrito^$^	7.2 ± 1.6	7.5 ± 1.7	7.2 ± 1.4	7.6 ± 1.6	7.3 ± 1.8	7.5 ± 1.7	0.585
	Tomatillo salsa verde burrito^$^	7.2 ± 1.8	7 ± 2	7.3 ± 1.7	7.5 ± 1.6	7 ± 1.8	6.5 ± 2.2	0.270
	Southwestern veggie bowl^$^	7.1 ± 1.6	7.2 ± 1.8	7 ± 1.7	7.6 ± 1.2	7.3 ± 1.5	6.7 ± 2.3	0.027
	Chicken apple sausage burrito^$^	6.8 ± 1.9	7.2 ± 1.9	7.1 ± 1.5	7.5 ± 1.5	6.5 ± 2.2	6.9 ± 2.3	0.936
	Tomatillo salsa verde bowl^$^	6.7 ± 1.9	6.8 ± 2	6.8 ± 1.9	7.3 ± 1.4	6.6 ± 2	6.1 ± 2.3	0.037
Dinners Entrees	Average rating across dinner entrees	7.1 ± 1.1	7 ± 1.7	7.1 ± 1.1	7.7 ± 1.1	7.1 ± 1.1	6.3 ± 1.9	< 0.001***
	Chicken teriyaki, brown rice, carrots	7.7 ± 1.1	7.5 ± 1.7	7.6 ± 1.2	8.3 ± 1	7.8 ± 1	6.7 ± 2	< 0.001***
	Chicken teriyaki, carrots, couscous	7.5 ± 1.5	7.3 ± 1.8	7.2 ± 1.6	7.8 ± 1.4	7.7 ± 1.3	6.7 ± 2	< 0.001***
	Chicken tikka masala, quinoa, and broccoli	7.3 ± 1.6	7.4 ± 1.8	7.5 ± 1.2	7.9 ± 1.2	7.2 ± 1.9	6.9 ± 2.1	0.150
	Chimichurri chicken, brown rice, cauliflower	7.2 ± 1.4	7.2 ± 1.9	7.1 ± 1.5	7.8 ± 1.1	7.3 ± 1.3	6.5 ± 2.3	< 0.001***
	Beef teriyaki, brown rice, cauliflower, bell pepper	7.2 ± 1.5	7.3 ± 1.9	7.3 ± 1.4	7.9 ± 1.2	7 ± 1.7	6.6 ± 2.3	0.056
	Chimichurri chicken, cauliflower, couscous	7.2 ± 1.5	7.1 ± 2.1	7.3 ± 1.3	7.9 ± 1.2	7.1 ± 1.6	6.1 ± 2.5	0.003
	Chicken tikka masala, red bell peppers, quinoa	7.2 ± 1.6	7.1 ± 1.9	7.1 ± 1.6	7.6 ± 1.4	7.2 ± 1.7	6.6 ± 2.3	0.012
	Tikka masala zoodles, cauliflower, broccoli, brown rice	7.1 ± 1.5	6.8 ± 1.9	6.9 ± 1.7	7.5 ± 1.3	7.3 ± 1.4	6 ± 2.2	< 0.001***
	Teriyaki pork, carrots, green beans, quinoa	7.1 ± 1.5	6.8 ± 2.1	6.9 ± 1.7	7.5 ± 1.3	7.4 ± 1.2	5.9 ± 2.5	< 0.001***
	Chimichurri beef, carrots, cauliflower, couscous	7.1 ± 1.6	7.1 ± 2	7.4 ± 1.6	7.8 ± 1.5	6.9 ± 1.6	6.3 ± 2.2	0.032
	Chimichurri zoodles, red bell peppers, brown rice	7 ± 1.7	6.6 ± 1.9	7 ± 1.7	7.3 ± 1.4	7 ± 1.8	5.9 ± 2.1	0.001
	Chimichurri beef, green peas, red bell peppers, couscous	7 ± 1.8	6.7 ± 2.2	7.3 ± 1.5	7.4 ± 1.8	6.7 ± 2	5.9 ± 2.5	0.040
	Dijon zucchini noodles, cauliflower, red peppers, lentils	6.8 ± 1.8	6.6 ± 1.9	6.8 ± 1.8	7.1 ± 1.6	6.9 ± 1.9	6 ± 2.1	0.001
	Dijon pulled pork, green peas, carrots, quinoa	6.6 ± 1.9	6.7 ± 2.1	6.7 ± 2	7.5 ± 1.4	6.6 ± 1.9	5.9 ± 2.4	0.001

^$^ = The dollar sign indicates meals we had to purchase more of at the end of the study; Rating = 9-point bipolar hedonic liking scale, 1 = Dislike extremely, 9 = Like extremely, question order randomized to reduce risk of order effect; *P*-values shown are the original *p*-values before Bonferroni correction, however, assessing by each meal leads to 28 comparison tests and high risk for Type 1 error. Therefore, Bonferroni adjusted α is set at 0.00179 for each individual meal, meaning the null hypothesis should only be rejected if the *p*-value is < 0.00179. *** = significant at *p* < .001

Clients in both groups had improvements in perceived diet quality from baseline to follow-up (ηp²=0.36, F(1,64) = 36.38, *p* < .001), but there were no between group differences or significant interactions (Fig. 2B). Clients in both groups had improvements in food security over time (ηp²=0.36, F(1,64) = 36.38, *p* < .001), but there were no between group effects or group by time interactions (Fig. 2C).

### Intervention satisfaction

Both groups reported high levels of satisfaction with their assigned interventions at follow-up. Participants in the meal kit group had higher satisfaction ratings than participants in the no-prep group (ηp²=0.09, F(1,64) = 6.28, *p* = .015). Table 4 displays these findings by group.

**Table 4:** Intervention Satisfaction, Client Meal Supplementation, and Perceived Entrée Size

	Across participants (N = 66)		Group 1 Meal Kits (N = 35)		Group 2 No-prep (N = 31)		Difference between groups
**Intervention satisfaction**	16.56	2.60	17.29	2.31	15.74	2.70	0.015*
**Added extra seasoning**							0.727
Yes	44(67%)		24(69%)		20(65%)		
No	22(33%)		11(31%)		11(35%)		
**Added extra food**							0.993
Yes	17(26%)		9(26%)		8(26%)		
No	49(74%)		26(74%)		23(74%)		
**Added extra snacks**							0.896
Yes	25(38%)		13(37%)		12(39%)		
No	41(62%)		22(63%)		19(61%)		
**Breakfast entrée size**							0.385
Not enough food	15(23%)		6(17%)		9(29%)		
The right amount of food	48(73%)		28(80%)		20(65%)		
Too much food	3(5%)		1(3%)		2(7%)		
**Dinner entrée size**							< 0.001***
Not enough food	8(12%)		0(0%)		8(26%)		
The right amount of food	57(86%)		35(100%)		22(71%)		
Too much food	1(2%)		0(0%)		1(3%)		

Values are n(%) for categorical variables and mean ± standard deviation for continuous variables; *=*p* < .05, **=*p* < .01, ***=*p* < .001

### Intervention fidelity

Of the 35 participants in the meal kit group, 33 indicated that they used the recipe cards provided to prepare meals (94%). Out of the 66 participants that completed the follow-up appointment and questionnaire, 67% indicated they added extra seasoning or condiments to the study meals (n = 44), 26% added extra food to the meals (n = 17), and 39% ate extra snacks in addition to the meals (n = 25). When asked about the size of the breakfast entrees provided, 73% of clients felt it was the right amount of food (n = 48), 23% felt it was not enough food (n = 15), and 5% thought it was too much food (n = 3). Regarding dinner entrees, 86% of clients felt it was the right amount of food (n = 57), 12% felt dinners did not provide enough food (n = 8), and only one participant (2%) felt dinners were too large. Clients in the no-prep group were more likely to indicate dinners did not provide enough food than clients in the meal kit group (Fisher’s exact test, *p* < .001). Table 4 displays these findings by group.

### Intervention costs

We anticipated providing 2,940 meals per group across the study period (35 participants, two meals per day, three people in the household, 14-days), therefore expected costs are presented as though all 2,940 meals were selected and actual costs are presented based on how many meals were selected. Across the two grants that funded this project, we budgeted $13,000 for meal kit supplies and ingredients or $4.42 per meal. Supplies (e.g., recyclable bags, printed recipes) for bundling the meal kits totaled $174.86 USD. Groceries cost $5,229.34 USD for a total expenditure of $5,404.20 USD. 2,106 servings were selected in the meal kit group across participants, for an actual total cost of $2.57 per meal. Without a donation from the no-prep meal distributor, expected costs were $20,580.00 USD or $7.00 per meal. With a donation of 50% of the initial 2,940 meals, expected costs at the outset of the study were $10,290.00 USD or $3.50 per meal. Replenishments of popular no-prep meals were purchased in the last three-weeks of the study for an additional $1,932 USD, and expected costs rose to $12,222.00 USD or $4.16 USD per meal. 2,296 no-prep meals were selected, therefore actual costs were $5.32 per meal. Replenished meals are indicated with a dollar sign symbol in Table 3. Intervention costs are summarized in Table 5.

**Table 5:** Intervention Costs

	Group 1 (Meal kits)		Group 2 (No-prep meals)	
	Total	Per meal	Total	Per meal
Expected costs	$13,000.00	$4.42	$10,290.00*	$3.50
Actual costs	$5,404.20	$2.57	$12,222.00	$5.32

$=United states dollar; *Expected costs include donation of 50% of the initial 2,940 meals, typically meals cost $7.00 per meal if purchased directly from the distributor

## Conclusions

In this pilot randomized controlled trial that provided food pantry clients with 14-days of nutritious meal kits (Group 1) or no-prep meals (Group 2) we found that hedonic liking for study meals was high and there were high intervention satisfaction ratings for both nutrition interventions. However, there was a between-within interaction on hedonic liking, such that participants that received meal kits had an increase in hedonic liking of study meals from baseline to follow-up, while participants that received no-prep meals had a slight decrease in hedonic liking. Similarly, participants in the meal kit group had higher intervention satisfaction ratings than participants in the no-prep group at follow-up.

Two prior studies have shown that people may enjoy healthy food more when self-prepared based on the hypothesis that exerting effort to prepare a meal increases the subjective value of the meal [28, 29]. Additionally, one consumer research study has shown no-prep meals have lower hedonic liking scores than homecooked meals, but that familiarity and sensory qualities of the meal (e.g., appearance, meal texture) can positively or negatively impact liking [30]. Further research on how familiarity (e.g., taste tests) and sensory improvements (e.g., improved packaging, better mouth feel) can be harnessed to improve hedonic liking of no-prep meals as well as study on how meal kits might increase hedonic liking and satisfaction is warranted.

There were some indications that, compared to meal kits, providing no-prep meals as chronic disease prevention and chronic disease management may not be as effective and desirable for this food pantry population (e.g., higher rates of dropout in the no-prep group, lower intervention satisfaction in the no-prep group, higher proportion of no-prep participants found dinners too small). Larger and longer studies are needed before that conclusion can be drawn given that hedonic liking and satisfaction ratings were generally favorable. Furthermore, it is possible that no-prep meals may have additional advantages for certain subsets of pantry populations, such as those with functional limitations or lack of kitchen space and equipment.

There were no between group differences or between-within interactions on perceived diet quality or food security that would suggest one approach was more effective than the other over the study period. There were large within group effects, showing that participants in both groups had significant improvements in food security and perceived diet quality over the study period. Follow-up studies are needed to see if improvements can be sustained over longer periods of time. Our preliminary hypotheses were that no-prep meals would be more effective at improving intervention satisfaction, diet quality, and food security. In this pilot study we did not find evidence supporting these hypotheses, however, given the small sample size, Type 2 error is possible.

### Strengths

The major strengths of this study were the randomized design, the ability to assess changes from baseline to follow-up, and our comprehensive measurement of food pantry clients’ preferences for two innovative nutrition interventions. Another major strength is our diverse population. Almost all participants (94%) were African American or Black or Hispanic or Latino, minoritized racial/ethnic groups historically underrepresented in research and most impacted by diet-related health inequities [31]. Additionally, 43% of participants (n = 30) completed all study procedures in Spanish and identified Spanish as their preferred language. A lack of representation of Hispanic or Latino participants and specifically Spanish speakers has been identified as a major limitation of community nutrition interventions [1, 32] and is specifically highlighted as a major limitation of a large ongoing trial comparing medically tailored meals (a type of no-prep meal) and meal vouchers for people with food insecurity [33]. Understanding which interventions are most desirable and efficacious for racially and ethnically minoritized populations, is imperative given that (1) these populations are most impacted by diet-related disease and (2) research has shown diet quality may improve for non-Hispanic clients after visiting a food pantry, but may stay the same for Hispanic or Latino clients, which suggests visiting a food pantry alone may not be sufficient to improve diet quality [1, 5], and that there may be disparities for Hispanic or Latino clients [34].

In our study we found that 47% of pantry clients did not have health insurance and 47% did not seek medical care at some point within the past two years due to concerns around cost. Therefore, providing meal kits and no-prep meals through traditional avenues, such as through food retailers, medical insurers, or by medical referral may not reach a large portion of people with food insecurity. A strength of our study was the ability to provide these innovative nutrition intervention strategies to clients in a location they already frequent to procure food at no cost.

Another strength of our study was that it was designed to understand whether meal kits and no-prep meals are desirable to food pantry clients. Our results indicate which study meals have the highest hedonic liking scores and can help researchers select and provide nutritious food options that align with clients’ preferences. Prior research has shown that client choice, taste preference, and intervention satisfaction are key components of effective nutrition interventions [19]. This study provides novel preliminary support that meal kits and no-prep meal interventions may be well-liked by pantry clients and provides insight on which meals to prioritize serving in future studies with pantry clients.

### Limitations

The major limitations of our study include the small sample size, which may have been underpowered to detect a between group effect or a between-within interaction. Systematic reviews that assess nutrition interventions in food pantries suggest it may take up to three-months to see differences in diet quality and up to six-months to see differences in clinical outcomes (e.g., HbA1c) [35, 36], therefore the short follow-up period is also a limitation.

Effects observed on food security and perceived diet quality may be caused by the provision of additional food outside of our study rather than the nutritional quality of the study foods. We could not find a study that explored whether providing extra food in addition to typical pantry offerings led to greater increases in food security, perceived dietary quality, or related outcomes (e.g., objective dietary quality, clinical outcomes), but is an option to consider as an active comparator group in the future.

Finally, we describe costs associated with implementing these interventions for this study but did not examine the comparative cost-effectiveness of the interventions. Given that there are different economies of scale in preparation of no-prep meals and meal kits, as well as different ways in which these processes can be integrated into food pantry operation at scale, these analyses were outside the scope of the pilot RCT. In this study, we purchased no-prep meals from a nutrition-focused meal distributor and purchased food for meal kits from a local grocery store. A more cost-effective way to implement in the future could be to use the pantries commercial kitchen to repurpose food donations into no-prep meals and meal kits, which could also have sustainability benefits if food nearing expiration could be used to create no-prep heat-and-eat meals. Having the pantry purchase ingredients that may not be consistently available through donations but needed to make the meal kits (e.g., spices, oils, eggs), directly at wholesale cost instead of buying in-store at retail cost could also save money and time. As the evidence base for no-prep meals and meal kits is more firmly established, cost-effectiveness and innovations in the integration of these programs into pantry operations will become an increasingly impactful area of inquiry.

Our study provides preliminary evidence that meal kits and no-prep meals were feasible to implement in a food pantry, well-liked by clients, and may increase food security and perceived diet quality among clients in the short-term. Future studies should continue to test both interventions as strategies to improve food security and dietary quality and include larger samples that allow for determining potential confounders or moderators. Studies should also include longer follow-up and objective measures of dietary quality and diet-related disease outcomes (e.g., HbA1c, blood pressure). While we included some preliminary implementation outcomes in the present study (e.g., acceptability, feasibility, fidelity), it is imperative to measure and compare intervention cost effectiveness and sustainability to ensure funds and efforts are being used effectively and able to be implemented on a wider scale [19].

### Electronic supplementary material

Below is the link to the electronic supplementary material.

**Additional file 1: Supplementary Material 1**. Study Protocol and Procedures. **Supplementary Material 2**. Meal kit ingredients. **Supplementary Material 3**. Servings Selected by Household Size

## Data Availability

The data used for the current analysis are available from the corresponding author upon reasonable request.
