# Supplementary material for: A pilot randomized controlled trial comparing nutritious meal kits and no-prep meals to improve food security and diet quality among food pantry clients
Source: BMC Public Health. 2023 Dec 1;23:2389. doi: 10.1186/s12889-023-17355-3 (PMC10691040; doi:10.1186/s12889-023-17355-3)
Supplement: Supplementary file 1 — Additional file 1: Supplementary Material 1. Study Protocol and Procedures. Supplementary Material 2. Meal kit ingredients. Supplementary Material 3. Servings Selected by Household Size [file 12889_2023_17355_MOESM1_ESM.docx]

**Supplementary Materials Table of Contents**

1. Study Protocol and Procedures
2. Meal Kit Ingredients
3. Table of Servings Selected by Household Size

Supplementary Material 1. Study Protocol and Procedures

| Screening and determination of eligibility | |
| --- | --- |
| 1. | Study staff sit at a table with study signage in food pantry waiting room. Clients approach table to learn more about the study. If no one is approaching, staff make an announcement “*We are doing a study to learn more about preferences for different nutrition programs and foods to bring into the pantry in the future. Please let us know if you’d like to learn more and complete a brief survey to see if you’re eligible to participate.”* |
| 2. | Client completes 10-item eligibility screener on a study tablet or by scanning QR code and completing on their personal device. If client prefers, study staff ask questions out loud and enter responses.  **Inclusion Criteria**: Adults 18 or older; able to read, write, and/or speak English or Spanish fluently; able to provide informed consent; used Crossroads food pantry at least once; able and willing to participate in the study, willing to return to the pantry in two-weeks to complete the follow-up appointment.  **Exclusion criteria**: Dietary restrictions, allergies, or sensitivities that could put the participant at-risk of harm from consuming study foods. |
| 3. | Staff check eligibility.  **If client is eligible**: Client is invited to participate and asked if they’d like to participate that day or schedule the baseline appointment for a future date.  **If client is ineligible**: Client is thanked for their interest and asked if they’d like to participate in a separate cross-sectional survey study, the study team was conducting concurrently. |
| Consent and baseline appointment | |
| 4. | Participant is brought to private office or cubicles onsite at the pantry. Participant is read consent form out loud by study staff in the participants preferred language (English or Spanish). Participant is asked if they have any questions, if they fully understand the study, and if they’re willing to participate. Staff record date and time of verbal consent in Excel spreadsheet. |
| 5. | Participant completes baseline questionnaire independently on study tablet or if they are uncomfortable with reading or using a tablet, staff reads questions out loud and enters responses. Group randomization is programmed into the questionnaire, such that the participant answers hedonic liking questions according to their group assignment (e.g., sees pictures of foods that correspond to their group). The order participants see study meals is randomized to reduce the risk of an order effect. |
| 6. | Participant becomes aware of randomization once asked to make food selections as concealment is no longer possible. Participant makes food selections using printed and laminated cards with names and pictures of study meals (participants in group 1 select from meal kit cards, participants in group 2 select from no-prep meal cards), staff enters selections into Excel spreadsheet. Spreadsheet is coded to alert the staff member if they have entered too many food selections. |
| 7. | Participant is scheduled for follow-up appointment for 14-days from baseline appointment date (or as close to that date as possible). Follow-up appointment is logged on study calendar. Participant is given a contact card with follow-up appointment date, day of the week, time, and study team contact information. |
| 8. | Participant is brought to semi-private cubicles to go through **the pantry’s usual order process (this was not manipulated – all participants received their usual services regardless of group)**. In the usual order process, clients select pantry foods with a pantry staff or volunteer team member on a computer that uses real-time Salesforce inventory software. Clients can visit the pantry once per month and can select food for up to 21 meals per person in their household based on a dietician developed algorithm. After clients select foods on the computer, their list is printed, and they walk through the aisles of the pantry which is set-up like a grocery store.  While clients are selecting food with pantry staff, study staff retrieve their study meals from the freezer (no-prep group) or bundle ingredients together with a recipe in a recyclable bag (meal kit group).  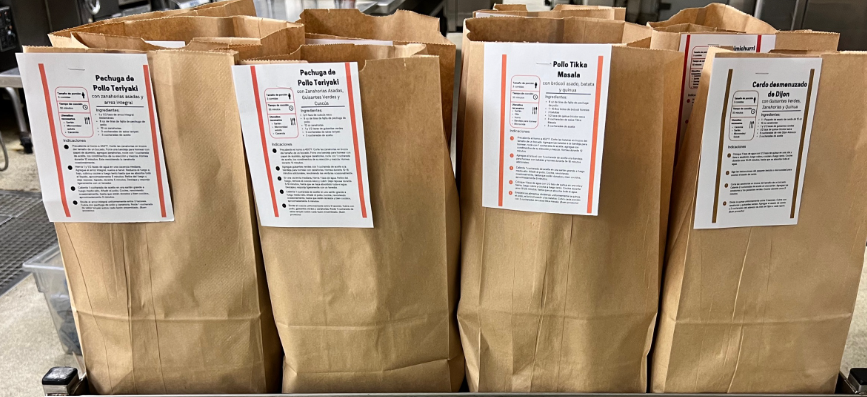  The list of ingredients used to make the meal kits is in Supplementary Material 2 below. |
| 9. | After packing participants food, study staff or student research assistants provide the participant with their study selections and if the participant would like, help them load their vehicle. |
| 10. | The participant is thanked for their time and reminder to contact us with any questions and return in two-weeks for their follow-up appointment. |
| At home (between baseline and follow-up appointments) | |
| 10. | Clients and members of their household are instructed to eat study meals over the study period. |
| 11. | Clients receive a reminder phone call, text, or email depending on their indicated preference the day prior to follow-up appointment. |
| Follow-up appointment | |
| 12. | Participant completes follow-up questionnaire independently on study tablet or if they are uncomfortable with reading or using a tablet, staff reads questions out loud and enters participant responses. |
| 13. | Participant is paid using a Greenphire ClinCard MasterCard per UT Southwestern Medical Center institutional policy. ClinCards can be used anywhere MasterCard is accepted, including to withdraw cash from an ATM. The ClinCard system requires a social security number, or the participant can choose to pay a 24% tax on the study payment upfront and not provide their social security number. This is clearly explained in the consent form and again during the follow-up appointment. |
| 14. | Participant is thanked for their time and walked to the exit. |

Supplementary Material 2. Meal kit ingredients

| Meal name (Meals were the same across groups) | Meal kit Ingredients (Matched to no-prep meal ingredients) |
| --- | --- |
| **Breakfast Entrees** |  |
| Turkey Sausage Burrito (with eggs) | Refrigerated turkey sausage roll (low sodium), dry carb balance tortilla, refrigerated egg whites, frozen pepper and onion blend |
| Southwestern Veggie Burrito (with egg whites) | Canned black beans (low sodium), dry carb balance tortilla, refrigerated egg whites, frozen pepper and onion blend, dry Southwest seasoning blend |
| Chicken Apple Sausage Burrito (with egg whites) | Frozen chicken and herb breakfast sausage, dry carb balance tortilla, refrigerated egg whites, fresh gala apple |
| Spinach Scramble Burrito (with egg whites) | Refrigerated egg whites, frozen spinach, frozen pepper and onion blend, dry carb balance tortilla |
| Tomatillo Salsa Verde Burrito (Plant and egg whites) | Refrigerated egg whites, dry carb balance tortilla, salsa verde sauce, frozen pepper and onion blend |
| Fire Roasted Salsa Scramble Burrito (Plant and egg whites) | Refrigerated egg whites, dry carb balance tortilla, canned diced tomatoes and green chilies (low sodium), canned black beans (low sodium), frozen spinach |
| Three Cheese Flatbread Single | Refrigerated egg whites, dry whole wheat pita bread, refrigerated reduced fat fiesta cheese blend, frozen pepper and onion blend |
| Spinach and Mozzarella Flatbread Single | Refrigerated egg whites, dry whole wheat pita bread, refrigerated reduced fat mozzarella cheese, frozen spinach, fresh sweet potato |
| Southwestern Veggie Bowl (with egg whites) | Canned black beans (low sodium), refrigerated liquid egg whites, frozen pepper and onion blend, dry Southwest seasoning blend, fresh russet potato, refrigerated reduced fat fiesta cheese blend |
| Turkey Sausage Bowl (with egg whites) | Refrigerated turkey sausage roll (low sodium), refrigerated liquid egg whites, frozen pepper and onion blend, fresh russet potato, refrigerated reduced fat mozzarella cheese |
| Tomatillo Salsa Verde Bowl (Plant & Egg White protein) | Canned black beans (low sodium), refrigerated liquid egg whites, frozen pepper and onion blend, salsa verde sauce, dry tri-color quinoa |
| Buttermilk Waffles | Dry whole wheat flour, refrigerated liquid egg whites, packaged honey, unsweetened almond milk, packaged apple sauce |
| Blueberry Waffles | Dry whole wheat flour, refrigerated liquid egg whites, packaged honey, unsweetened almond milk, frozen blueberries, packaged apple sauce |
| Cinnamon French Toast | Dry whole wheat bread, refrigerated liquid egg whites, unsweetened almond milk, dry cinnamon powder, vanilla extract |
| **Dinner Entrees** |  |
| Tikka Masala Chicken with Red bell Pepper, Roasted Sweet Potato, and Tri-Color Quinoa | Tikka masala sauce, precooked chicken breast fajita strips, frozen pepper and onion blend, fresh sweet potatoes, dry tri-color quinoa |
| Chimichurri Chicken with Couscous and Roasted Cauliflower | Chimichurri sauce, precooked chicken breast fajita strips, frozen cauliflower, dry couscous |
| Dijon Zucchini Noodles with Cauliflower, Red Peppers, and Lentils | Honey Dijon dressing (no sugar added), frozen zucchini spirals, frozen cauliflower, dry lentils, frozen pepper and onion blend |
| Teriyaki Pork with Roasted Carrots, Green Beans, and White Quinoa | Teriyaki sauce, precooked pork in au jus and savory sauce, fresh carrots, frozen green beans, dry white quinoa |
| Tikka Masala Chicken with Roasted Broccoli, Sweet Potato, and Quinoa | Tikka masala sauce, precooked chicken breast fajita strips, frozen broccoli, fresh sweet potatoes, dry tri-color quinoa |
| Chimichurri Beef with Green Peas, Roasted Red Peppers, and Couscous | Chimichurri sauce, precooked steak fajita strips, frozen pepper and onion blend, frozen sweet peas, dry couscous |
| Teriyaki Chicken Breast with Roasted Carrots, Green Peas, and Couscous | Teriyaki sauce, precooked chicken breast fajita strips, fresh carrots, frozen sweet peas, dry couscous |
| Chimichurri Chicken with Roasted Cauliflower, Carrots, and Brown Rice | Chimichurri sauce, precooked chicken breast fajita strips, frozen cauliflower, fresh carrots, dry brown rice |
| Tikka Masala Zoodles with Roasted Cauliflower, Broccoli, and Brown Rice | Tikka masala sauce, frozen zucchini spirals, frozen cauliflower, frozen broccoli, brown rice |
| Chimichurri Beef with Carrots, Roasted Cauliflower, and Couscous | Chimichurri sauce, precooked steak fajita strips, fresh carrots, frozen cauliflower, dry couscous |
| Teriyaki Chicken with Sweet Corn, Green Peas, and Brown Rice | Teriyaki sauce, precooked chicken breast fajita strips, frozen sweet peas, frozen corn kernels, dry brown rice |
| Chimichurri Zoodles with Red Bell Pepper, Roasted Cauliflower, and Brown Rice | Chimichurri sauce, frozen zucchini spirals, frozen pepper and onion blend, dry brown rice |
| Dijon Pulled Pork with Green Peas, Carrots, and Tri-color Quinoa | Honey Dijon dressing (no sugar added), precooked pork in au jus and savory sauce, fresh carrots, frozen sweet peas, dry tri-color quinoa |
| Teriyaki Beef with Cauliflower, Red Bell Pepper, and Brown Rice | Teriyaki sauce, precooked steak fajita strips, frozen cauliflower, frozen pepper and onion blend, dry brown rice |
| Notes: 1) Meal kits and meal kit ingredients were matched as closely as possible to no-prep meal ingredients; 2) Regardless of food selections, all meal kit participants received one of each of the following ingredients as they were more affordable to provide in bulk and were used in multiple recipes: 64 oz canola oil, 2 oz vanilla extract, 2.5 oz ground cinnamon, 32 oz shelf-stable unsweetened almond milk | |

Supplementary Material 3. Servings Selected by Household Size

| Household size | All participants | | | Group 1 Meal Kits | | | Group 2 No-prep Meals | | | p-value |
| --- | --- | --- | --- | --- | --- | --- | --- | --- | --- | --- |
|  | N | Servings Selected | | N | Servings Selected | | N | Servings Selected | |  |
|  |  | Mean | SD |  | Mean | SD |  | Mean | SD |  |
| 1 | 14 | 61.14 | 22 | 8 | 55.88 | 23.12 | 6 | 68.17 | 20.15 |  |
| 2 | 11 | 56.27 | 19.27 | 4 | 61.5 | 26.10 | 7 | 53.29 | 15.80 |  |
| 3 | 5 | 51.6 | 15.79 | 1 | 51 | . | 4 | 51.75 | 18.23 |  |
| 4 | 15 | 64.93 | 17.92 | 11 | 62.73 | 19.74 | 4 | 71 | 11.52 |  |
| 5 | 15 | 66.2 | 19.45 | 6 | 60.5 | 17.64 | 9 | 70 | 20.66 |  |
| 6 | 5 | 70.2 | 22.01 | 3 | 69 | 28.62 | 2 | 72 | 16.97 |  |
| 7 | 3 | 74 | 17.32 | 1 | 54 | . | 2 | 84 | 0 |  |
| 10 | 1 | 81 | . | 0 | . | . | 1 | 81 | . |  |
| 12 | 1 | 48 | . | 1 | 48 | . | 0 | . | . |  |
| Total servings | 70 | 62.89 | 19.38 | 35 | 60.17 | 20.01 | 35 | 65.60 | 18.62 | .244 |
| Servings per person* | 70 | 25.66 | 21.54 | 35 | 25.10 | 21.47 | 35 | 26.22 | 21.92 | .829 |
| Servings per person = number of servings selected divided by number of people in the household | | | | | | | | | | |
